# Regulation of flowering time in chrysanthemum by the R2R3 MYB transcription factor *CmMYB2* is associated with changes in gibberellin metabolism

**DOI:** 10.1038/s41438-020-0317-1

**Published:** 2020-07-01

**Authors:** Lu Zhu, Yunxiao Guan, Yanan Liu, Zhaohe Zhang, Muhammad Abuzar Jaffar, Aiping Song, Sumei Chen, Jiafu Jiang, Fadi Chen

**Affiliations:** State Key Laboratory of Crop Genetics and Germplasm Enhancement, Key Laboratory of Landscaping, Ministry of Agriculture, College of Horticulture, Nanjing Agricultural University, Nanjing, China

**Keywords:** Transgenic organisms, Transcriptomics

## Abstract

The switch from vegetative growth to reproductive growth is a key event in the development of a plant. Here, the product of the chrysanthemum gene *CmMYB2*, an R2R3 MYB transcription factor that is localized in the nucleus, was shown to be a component of the switching mechanism. Plants engineered to overexpress *CmMYB2* flowered earlier than did wild-type plants, while those in which *CmMYB2* was suppressed flowered later. In both the overexpression and RNAi knockdown plants, a number of genes encoding proteins involved in gibberellin synthesis or signaling, as well as in the response to photoperiod, were transcribed at a level that differed from that in the wild type. Both yeast two-hybrid and bimolecular fluorescence complementation assays revealed that CmMYB2 interacts with CmBBX24, a zinc-finger transcription factor known to regulate flowering by its influence on gibberellin synthesis.

## Introduction

The switch from vegetative growth to reproductive growth is a major developmental event in the life cycle of a flowering plant. This switch is coordinated by the products of a suite of genes that differentially respond to the photoperiod, temperature, and the tissue content of several phytohormones^1^. The successful isolation of a number of such genes has led to the recognition that flowering can be triggered by proteins active in one or more of the photoperiod, gibberellin (GA), vernalization, autonomous, and senescence pathways^2–6^. These pathways come together to form a complex regulatory network that ultimately induces the key floral meristem genes *APETALA1* (*AP1*) and *LEAFY* (*LFY*)^4–6^.

A number of MYB transcription factors participate in the regulation of flowering time. The following examples all relate to the model angiosperm species *Arabidopsis thaliana*. Plants engineered to overexpress the *MYB*-related gene *CIRCADIAN CLOCK ASSOCIATED 1* (*CCA1*) are compromised with respect to their recognition of the circadian clock and consequently suffer from a delay in flowering^7^. Overexpression of the gene *EARLY-PHYTOCHROME-RESPONSIVE* 1 (*EPR*1) enhances responsiveness to far-red light, which also leads to delayed flowering^8^. Overexpression of *CAPRICE 3* (*CPL3*) downregulates several key flowering time genes, including *FLOWERING LOCUS T* (*FT*), *SUPPRESSOR OF OVEREXPRESSION OF CO1* (*SOC1*) and *CONSTANS* (*CO*)^9^. The product of *WEREWOLF* (*WER*) is known to act in the photoperiod pathway, since flowering is delayed in the *wer* mutant when exposed to long-day conditions^10^. MYB30 interacts with the *FT* promoter to promote flowering^11^. EARLY FLOWERING MYB PROTEIN (EFM) is involved in both the photoperiod and the temperature flowering pathways^12^, while PHLOEM DEVELOPMENT (APL) (syn. PE) activates *FT*^13^. The flowering of plants overexpressing *MYB44* is delayed by 6–7 days^14^. MYB transcription factors that affect flowering are also present in other species. For example, the product of the poplar gene *PtrMYB192* negatively affects flowering time, and its constitutive expression in *A*. *thaliana* also induces a delay in flowering^15^. Constitutive expression of the wheat gene *TaMYB72* in rice delays flowering time by 12 days^16^. In alfalfa, MsSPL13 downregulates *MsMYB112*, thereby controlling both vegetative and reproductive development^17^. In chrysanthemum (*Chrysanthemum morifolium*), a substantial number of MYB transcription factors have been shown to participate in the determination of flowering time^18,19^, although little is known about their specific functions.

Chrysanthemum occupies a large share of the global market of cut flowers^20,21^. Most chrysanthemum varieties are short-day plants and blossom during a relatively short period. The *CmMYB*2 gene encodes an R2R3 MYB transcription factor; when this gene is constitutively expressed in *A*. *thaliana*, flowering is delayed, and all *CO*, *FT*, *SOC*1, *LFY*, and *AP*1 genes are downregulated^22^. However, in this study, when *CmMYB2* is overexpressed in chrysanthemum, the flowering time is promoted, while RNAi-enabled knockdown delays flowering. The regulatory mechanism of flowering was further analyzed via transcriptome sequencing. CmMYB2 was found to interact with CmBBX24, which is a zinc-finger transcription factor that has been shown to alter GB synthesis and regulate flowering time^23^. Thus, the aim of the present study was to characterize in detail the molecular effect of manipulating the expression of *CmMYB*2. The results showed that *CmMYB*2 may regulate flowering by inhibiting the activity of the CmBBX24 protein and modulating the GA pathway in chrysanthemum.

## Results

### Subcellular localization and transactivation activity of CmMYB2

**Fig. 1:**
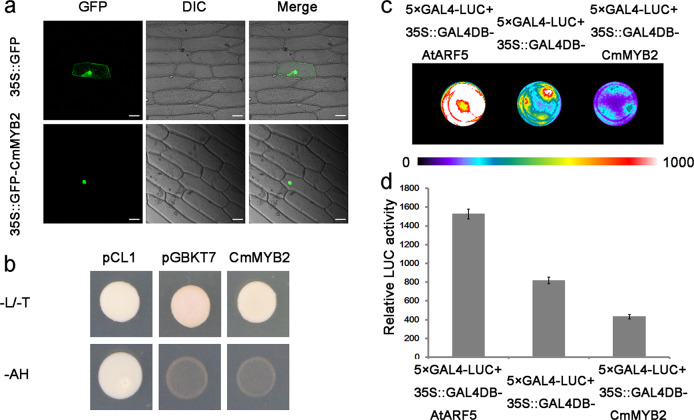
Subcellular localization and transactivation activity of *CmMYB2*. **a** Site of CmMYB2 deposition in transiently transformed onion epidermal cells. Bar: 50 μm. **b** Transcriptional activation activity of *CmMYB2* in yeast. pCL1 and pGBKT7 plasmids were used as the positive and negative controls, respectively. –L/-T: SD media lacking leucine/SD media lacking tryptophan, -AH: SD media lacking both histidine and adenine. **c**, **d** Luciferase activity in *A*. *thaliana* mesophyll protoplasts expressing 5×GAL4-LUC together with either p*35S::GAL4DB-AtARF5*, p*35S::GAL4DB* or p3*5S::GAL4DB-CmMYB2*. **c** Arabidopsis mesophyll protoplasts imaged after luciferin addition with a charge-coupled device (CCD) camera. **d** Luciferase activity measured after the introduction of p*35S::GAL4DB-CmMYB2* into *A*. *thaliana* mesophyll protoplasts

The sequences of the *CmMYB2* open reading frame present in the varieties Jinba and Zhongshanzigui were identical stretches of 948 nt, predicted to encode a 315 residue polypeptide. The polypeptide sequence included an R2R3 MYB domain within its N-terminal region and the characteristic GxFMxVVQEMIxxEVRSYM motif within its C-terminal region^22^. The site of expression of *CmMYB*2 was deduced by transiently expressing a transgene comprising the *CmMYB2* coding sequence fused to *GFP* and driven by the CaMV 35S promoter in onion epidermal cells. GFP activity was concentrated in the nuclei, while in control transgenic plants harboring the p35*S::GFP* construct, it was dispersed throughout the nucleus and cytoplasm (Fig. 1a).

To determine whether CmMYB2 can function as a transcription factor, a transactivation assay was performed in yeast. Yeast cells harboring pCL1 were able to grow freely on synthetic dropout (SD) media lacking histidine and adenine, while cells harboring pGBKT7 were able to grow only on SD media lacking tryptophan. Yeast cells harboring *CmMYB2* were unable to activate either the *His3* or the *Ade2* reporter gene, preventing growth on SD media lacking histidine and adenine (Fig. 1b). The results suggested that CmMYB2 showed no transactivation activity in yeast. A series of equivalent assays was attempted in *A*. *thaliana* protoplasts, in which the positive control was p*35S::GAL4DB-ARF5* and in which the negative control was p*35S::GAL4DB*. The reduction in LUC activity exhibited by protoplasts harboring *CmMYB2* compared to the activity in protoplasts harboring p*35S::GAL4DB* suggested that CmMYB2 could act as a transcriptional repressor in *A*. *thaliana* protoplasts (Fig. 1c).

### Effects of manipulating the expression of *CmMYB*2 on plant growth and development

**Fig. 2:**
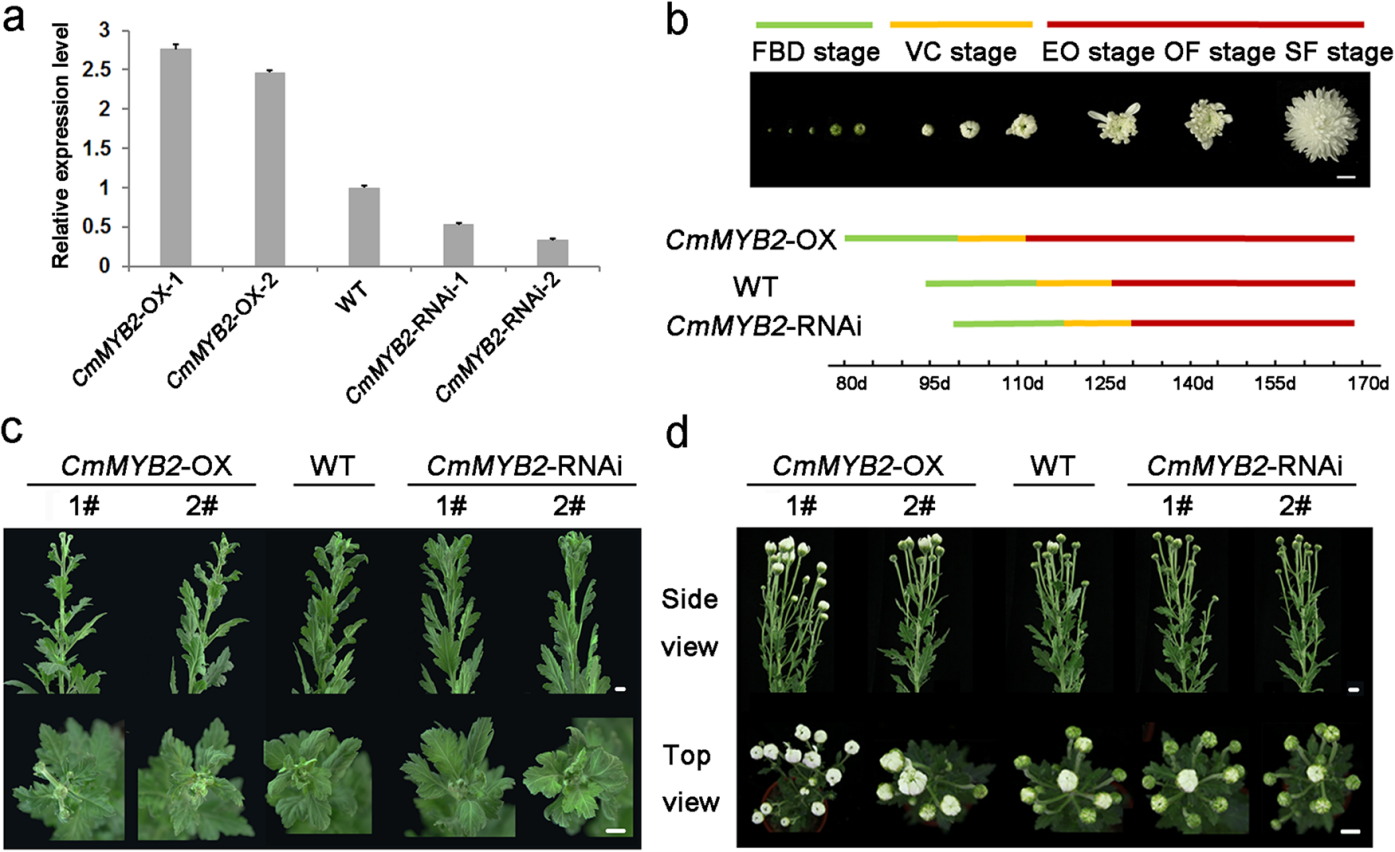
Phenotypic characterization of *CmMYB2*-overexpressing (OX) and CmMYB2 knockdown (RNAi) chrysanthemum plants. **a** qRT-PCR analysis of *CmMYB2* transcription in WT, OX and RNAi plants. OX-1, 2: two independent *CmMYB2* overexpressors, RNAi-1, -2: two independent *CmMYB*2 RNAi knockdowns. The values are shown as the mean ± SEs (*n* = 3). **b** Development of flower buds in transgenic and WT plants. FBD: flower bud development stage, VC: visible color stage, EO: early opening stage, OF: open-flower stage, SF: senescent-flower stage. Bars: 1 cm. **c**, **d** Phenotypes of WT, OX and RNAi plants at the reproductive stage. Plants were imaged at (**c**) 100 days and (**d**) 120 days after transplanting

Both *CmMYB*2 overexpression (OX lines) and *CmMYB*2 knockdown (RNAi lines) were generated, and their growth and development were monitored at various time points (Fig. 2a). The flowering time of both the OX and RNAi plants differed significantly from that of wild-type (WT) plants. At 80 days after transplanting, OX plants had formed visible flower buds and had entered the flower bud development stage (FBD); whereas this event occurred at 95 days in WT plants and at 99 days in RNAi plants. The differentiation of the first flower buds and beginning of the petal primordium differentiation stage occurred 15 days sooner in OX plants than in WT plants, while the RNAi plants took an additional four days to reach this stage. By 112 days after transplanting, the flowers of OX plants had entered the early opening stage (EO); however, those formed by WT plants did not reach this stage until 127 days after transplantation, and those formed by RNAi plants did not reach it until 130 days after transplantation (Fig. 2b–d). There were no significant differences among the OX, RNAi and WT plants with respect to their overall height, leaf and flower number or diameter of their flowers.

### Transcription profiling of genes acting downstream of *CmMYB*2 and associated with flowering time

**Fig. 3:**
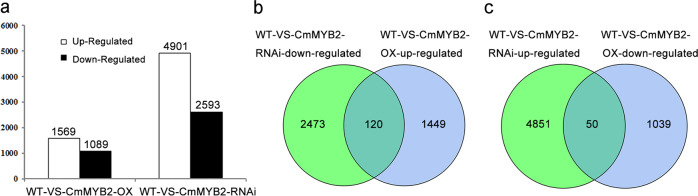
Unigenes transcribed differentially between WT and transgenic chrysanthemum. **a** Number of differentially transcribed genes in the comparisons WT *versus* OX and WT *versus* RNAi. **b**, **c** Venn diagram showing the set of differentially transcribed genes (**b**) upregulated in OX and downregulated in RNAi plants and (**c**) downregulated in OX and upregulated in RNAi plants

**Fig. 4:**
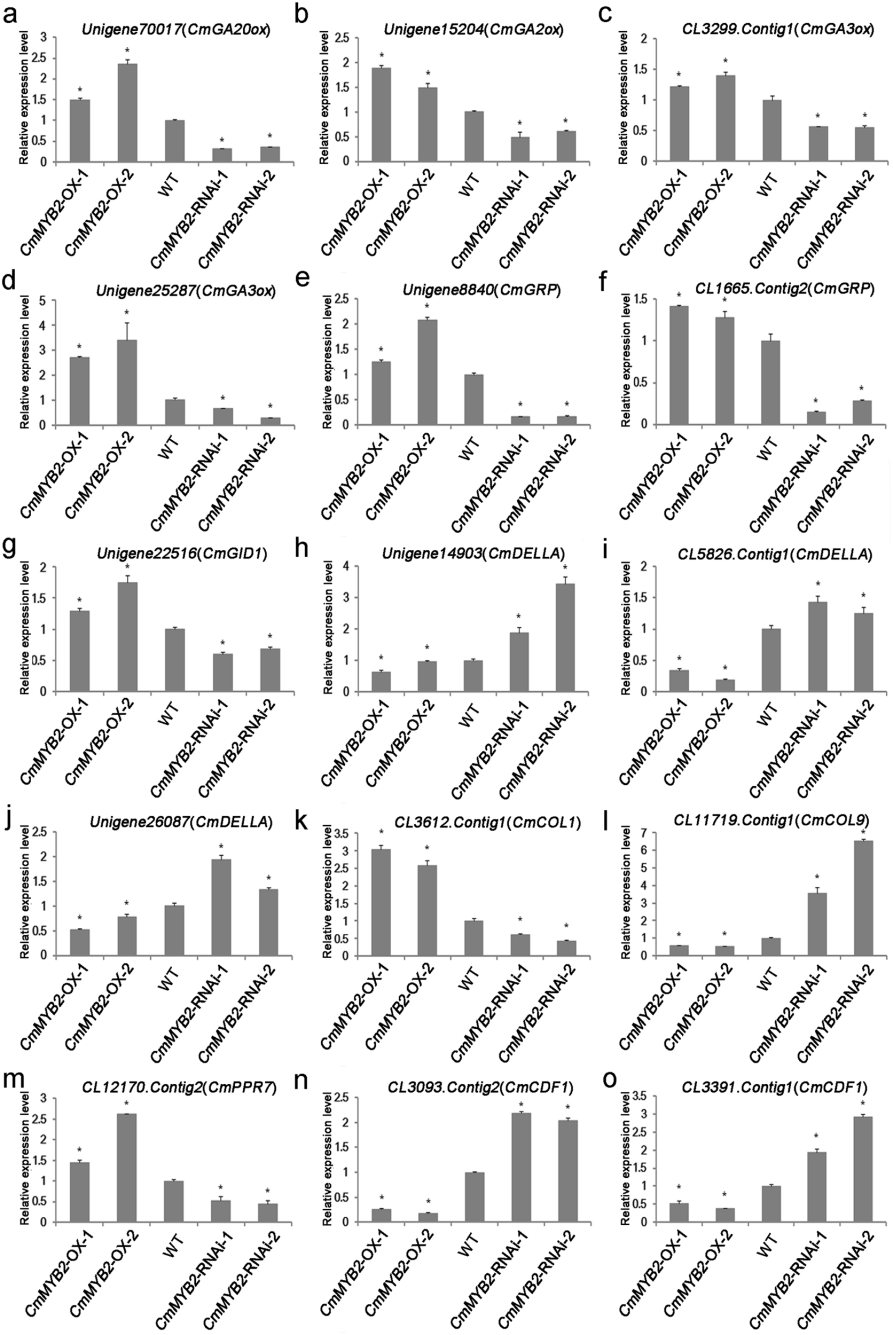
qRT-PCR validation of differential transcription predicted by RNA-seq. **a***CmGA20ox*, **b***CmGA*2*ox*, **c***CmGA3ox*, **d***CmGA*3*ox*, **e***CmGRP*, **f***CmGRP*, **g***CmGID1*, **h***CmDELLA*, **i***CmDELLA*, **j***CmDELLA*, **k***CmCOL1*, **l***CmCOL9*, **m***CmPRR7*, **n***CmCDF1*, **o***CmCDF1*. The values are shown as the mean ± SEs (*n* = 3). The means for each gene followed by the same letter do not differ significantly from one another (*P* < 0.05)

RNA-seq was implemented to identify differential transcription between WT, OX and RNAi plants (Fig. 3a); Venn diagrams were constructed to show the distribution of these genes visually (Fig. 3b, c). The genes that were either more strongly transcribed in the OX plants than in the WT plants but weakly transcribed in the RNAi plants or vice versa were emphasized. Among the large number of such genes identified, many of their encoded proteins were involved in either the GA synthesis/signaling pathway or the photoperiod pathway; of particular interest were *CmGA20ox*, *CmGA2ox*, *CmGA3ox*, *CmGRP*, *CmGID*, and *CmDELLA* (the GA synthesis and signaling pathway) as well as *CmCOL1*, *CmCOL9*, *CmPRR7*, *CmPRR9*, and *CmCDF1* (the photoperiod pathway) (Table 1). The results of the quantitative real-time PCR (qRT-PCR) assays used to confirm the RNA-seq-based identification are shown in Fig. 4. The analysis confirmed that *CmGA20ox* (Unigene70017), *CmGA2ox* (Unigene15204), *CmGA3ox* (CL3299.Contig1 and Unigene25287), *CmGRP* (Unigene8840 and CL1665.Contig2), *CmGID1* (Unigene22516), *CmCOL1* (CL3612.Contig1) and *CmPRR7* (CL12170.Contig2) were more strongly transcribed in OX plants than in WT plants but were more weakly transcribed in RNAi plants; moreover, the abundance of *CmDELLA* (Unigene14903, CL5826.Contig1 and Unigene26087), *CmCOL9* (CL11719.Contig1) and *CmCDF1* (CL3093.Contig2 and CL3391.Contig1) transcripts was lower in the OX plants than in the WT plants, but it was higher in the RNAi plants.

**Table 1:** Differentially transcribed genes associated with flowering time

Gene	Annotation	Fold Change (log2 transformed)	
		OX/WT	RNAi/WT
*GA biosynthesis and signaling*			
Unigene70017_All	GA 20-oxidase	1.0	−5.4
Unigene15204_All	GA 2-oxidase	0.7	−1.0
CL3299.Contig1_All	GA 3-oxidase 1-like	0.3	−1.5
Unigene25287_All	GA 3-oxidase 4	0.3	−2.3
Unigene8840_All	Gibberellin-regulated family protein	2.0	−0.7
CL1665.Contig2_All	Gibberellin-responsive protein	0.2	−4.6
Unigene22516_All	Gibberellin receptor GID1	0.6	−1.3
Unigene14903_All	DELLA protein	−0.3	1.9
CL5826.Contig1_All	DELLA protein	−3.5	0.3
Unigene26087_All	DELLA protein	−0.4	1.0
*Photoperiod pathway*			
CL3612.Contig1_All	CONSTANS-LIKE 1	1.6	−1.1
CL11719.Contig1_All	CONSTANS-LIKE 9	−1.1	1.5
CL12170.Contig2_All	PSEUDO-RESPONSE REGULATOR 7	0.6	−1.1
CL3093.Contig2_All	CYCLING DOF FACTOR 1	−1.2	0.6
CL3391.Contig1_All	CYCLING DOF FACTOR 1	−0.9	1.5

The thresholds for significance were *P* < 0.05 and FDR ≤ 0.001

*WT* wild type, *OXCmMYB2* overexpression, *RNAiCmMYB2* knockdown

### *CmMYB2* alters cellular GA contents through its interaction with *CmBBX*24

**Fig. 5:**
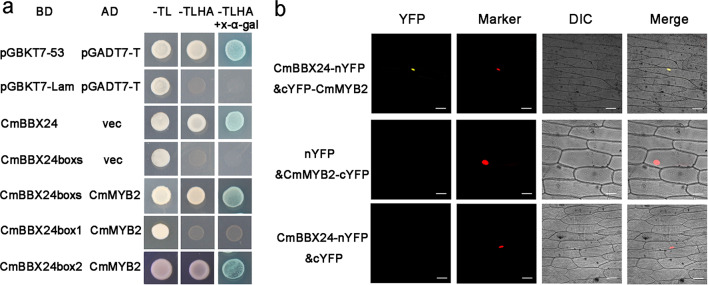
CmMYB2 interacts with CmBBX24 both in vitro and in vivo. **a** A yeast two-hybrid assay showing protein interactions. -TL, SD/-Trp/-Leu; -TLHA, SD/-Trp/-Leu/-Ade/-His; -TLHA + X-*α*-gal, SD/-Trp/-Leu/-Ade/-His/X-*α*-gal; vec, empty vector; CmBBX24boxs, two B-boxes in the N-terminus of *CmBBX24*; CmBBX24box1, the first B-box in the N-terminus of *CmBBX24*; CmBBX24box2, the second B-box in the N-terminus of *CmBBX24*; pGBKT7-53 and pGADT7-T served as positive controls, and pGBKT7-Lam and pGADT7-T served as negative controls. **b** A BiFC assay was used to confirm the interaction between CmMYB2 and CmBBX24, given the results of the yeast two-hybrid assay. YFP: yellow fluorescent protein, Marker: nuclear localization shown by RFP activity, DIC: bright field image, Merge: overlay of YFP, RFP and bright field images

Quantification of the GA_1_, GA_19_, and GA_20_ contents of young leaves of WT, OX and RNAi plants grown under short days showed that these compounds were significantly enhanced in OX plants and significantly reduced in RNAi plants (Table 2). A yeast two-hybrid screen was then performed to identify the proteins able to interact with CmMYB2. CmMYB9A was found to be a potential interacting protein with CmMYB2, and CmBBX24 was also found to interact with CmMYB9A (Table S2). This implied that CmMYB2 may directly interact with CmBBX24. *CmBBX24* contained two B-boxes at the N-terminus. *CmBBX24boxs* was two B-boxes at the N-terminus of *CmBBX24* and *CmBBX24box2* was the second B-box at the N-terminus of *CmBBX24*. Yeast cells cotransformed with *CmMYB2* and *CmBBX24boxs* were able to grow on media lacking tryptophan, leucine, histidine and adenine, regardless of whether X-*α*-gal was supplied (Fig. 5a). Additional experiments showed that CmMYB2 was able to interact with CmBBX24box2 (Fig. 5a). A bimolecular fluorescence complementation (BiFC) assay was used to provide experimental evidence that CmMYB2 could interact with CmBBX24 in vivo. In onion epidermal cells transiently transformed with *CmMYB2/cYFP* and *CmBBX24/nYFP* fusion constructs, YFP fluorescence was detected within the nuclei (Fig. 5b), confirming an in vivo interaction between CmMYB2 and CmBBX24.

**Table 2:** GA content in the leaves of WT and transgenic (OX and RNAi) chrysanthemum plants grown under short-day conditions

	GA content (ng g^−1^ FW)		
Lines	GA_1_	GA_19_	GA_20_
WT	2.78 ± 0.33	4.85 ± 0.74	10.41 ± 0.33
*CmMYB2*-OX-1	3.22 ± 0.41*	5.70 ± 0.92*	12.39 ± 0.19**
*CmMYB2*-RNAi-1	1.46 ± 0.16**	2.34 ± 0.32**	6.70 ± 0.48**

SD: 8 h photoperiod. The values are shown as the mean ± SEs (*n* = 3). * and ** represent significant differences (*P* < 0.05, <0.01). GA_4_, GA_9_ and GA_24_ were not detected

## Discussion

### CmMYB2 is expressed in the nucleus and acts as a transcriptional repressor

A common feature of MYB transcription factors is their nuclear localization signal peptides^24–26^. The rice gene *OsMYB*511 is expressed specifically in the nucleus^24^, as are the *Jatropha curcas* gene *JcR1MYB1*^25^ and the *Nicotiana benthamiana* gene *NbPHAN*^26^. Experiments have shown that for NbPHAN, the N-terminal region is responsible for its nuclear localization^26^. Here, a transient expression experiment based on onion epidermal cells was able to show that CmMYB2 also localizes to the nucleus, as would be expected for a gene product that controls the transcription of other genes. The full wheat TaMYB73 protein, as well as its C-terminal region, exhibits self-activating ability, but its N-terminal region does not^27^. Previous experiments have shown that the CmMYB19 protein exhibits no transcriptional activation activity when its encoding gene is expressed in yeast^28^. Here, the outcome of a yeast one-hybrid experiment demonstrated that CmMYB2 similarly failed to exhibit transcriptional activation, while its product was suggested to be able to act as a transcriptional repressor when the gene was expressed in *A*. *thaliana* protoplasts.

### Involvement of *CmMYB*2 in the control of flowering time

To transition from the vegetative to the reproductive state, higher plants need to integrate particular environmental signals (such as the photoperiod) with specific endogenous stimuli (such as GA)^29,30^. Both the photoperiod and the GA pathway, which strongly control the differentiation of flower buds, are highly conserved among plants and have been intensively studied^1,31^. Here, the RNA-seq approach used to profile the transcriptomes revealed that homologs of a number of known components of the various flowering regulatory pathways were identified as being differentially transcribed between WT plants and those in which the level of expression of *CmMYB2* was either increased or decreased; among these homologs were the GA synthesis/signaling genes *CmGA20ox*, *CmGA2ox*, *CmGA3ox*, *CmGRP*, *CmGID*, and *CmDELLA* and the photoperiod pathway genes *CmCOL1*, *CmCOL9*, *CmPRR7*, *CmPRR9*, and *CmCDF1*. The strong implication is that CmMYB2 influences flowering time in chrysanthemum by regulating GA and photoperiod pathway genes.

MYB transcription factors have a major impact on plant growth and development, but their contribution to the determination of flowering time is not well understood; much of the relevant data is related to *A*. *thaliana* and a small number of crop species. The *MYB*-related proteins *MYB-RELATED PROTEIN 1* (*MYR1*) and *MYB-RELATED PROTEIN 2* (*MYR2*) have been shown to be repressors of flowering in plants maintained under low-light intensity, since the *myr1/myr2* double mutant flowers earlier than WT does^32^. Moreover, the phenotype resulting from overexpression of *MYR1* and *MYR2* resembles that of a GA-deficient plant. Here, under short-day conditions, chrysanthemum plants engineered to overexpress *CmMYB2* flowered earlier than did WT plants, the latter of which in turn flowered before *CmMYB2* knockdown plants did. Altering the level of *CmMYB*2 expression affected the leaf content of three forms of GA (GA_1_, GA_19_, and GA_20_). *CmBBX24* knockdown plants also flower before WT plants, and the transcription of the *CmBBX24* gene is affected by the photoperiod; it can also be manipulated by the application of GA^23^. It was possible to show that CmMYB2 and CmBBX24 interacted both in vitro and in vivo. It is possible that *CmMYB*2 relieves the CmBBX24 inhibition of the GA pathway based on their interaction and reverse effects on flowering. Unexpectedly, the flowering time of *A*. *thaliana* plants constitutively expressing *CmMYB2* is delayed when plants are exposed to long-day conditions^22^. This difference in behavior may be related to the fact that, unlike the short-day plant species Jinba chrysanthemum, flowering of *A*. *thaliana* is induced by long days. Heterologous transformation of a gene leading to contrasting flowering times has also been reported in wheat and Arabidopsis^33^. The details of the mechanism thus need to be elucidated.

In conclusion, overexpression of the chrysanthemum gene *CmMYB2*, which encodes a MYB protein, accelerated flowering, while its suppression delayed flowering. CmMYB2 probably exerts its effect on flowering time through the GA pathway, since the leaf GA content was responsive to the intensity of *CmMYB2* expression. This mode of action is consistent with CmMYB2’s ability to interact with CmBBX24, because the latter protein is also known to regulate flowering through the GA pathway^23^.

## Materials and methods

### Plant materials and cultivation conditions

Cuttings of the chrysanthemum variety Jinba at the five- or six-leaf stage were obtained from Nanjing Agricultural University’s Chrysanthemum Germplasm Resource Preservation Center (Nanjing, China) and were grown in a 1:1:1 (v/v/v) nutrient-enriched soil:vermiculite:perlite mixture.

### Isolation of *CmMYB*2

A 1 μg aliquot of RNA extracted from the leaves of a Jinba plant using RNAiso reagent (TaKaRa, Tokyo, Japan) was converted into ss cDNA using M-MLV reverse transcriptase (TaKaRa). The full-length *CmMYB2* sequence of Jinba was inferred from the version of the sequence present in the variety Zhongshanzigui^22^. The full-length *CmMYB2* cDNA sequence was subsequently inserted into a pMD19-T easy vector (TaKaRa) for validation by sequencing. The relevant primers used for this procedure are listed in Table S1.

### Subcellular localization of CmMYB2

The *CmMYB2* open reading frame was amplified using the primer pair CmMYB2-pENTR1A-F/-R, which ensured that the amplicon carried a *Sal*I recognition site at its 5′ end and a *Not*I recognition site at its 3′ end (the primer details are listed in Table S1). The resulting amplicon was inserted into a pENTR1A vector (Invitrogen, Carlsbad, CA, USA) and subsequently into a pMDC43 vector^34^ using a reaction based on LR Clonase II (Invitrogen). The *CmMYB2* sequence was fused to the N-terminus of *GFP*, and the fusion construct was placed under the control of the CaMV 35 S promoter, forming a p*35S::GFP-CmMYB2* construct. This construct (or, as a control, p*35S::GFP*) was transformed into onion epidermal cells using a PDS-1000 He-driven particle bombardment device (Bio-Rad, Hercules, CA, USA). The bombarded tissue was cultivated in the dark at 23 °C for 16 h on Murashige and Skoog (MS) media^35^ before being assayed for GFP activity using confocal laser scanning microscopy (LSM780, Zeiss, Oberkochen, Germany).

### Analysis of the transcriptional activity of CmMYB2

The *CmMYB2* open reading frame was amplified using the primer pair MYB2-BD-F/-R (see Table S1 for details), which added an *Nde*I recognition site to the 5’ end and a *Bam*HI recognition site to the 3′ end. After purification, the amplicon was inserted into a pGBKT7 vector (Clontech, Mountain View, CA, USA). A pCL1 vector containing a full-length copy of *GAL4* served as a positive control, and an empty pGBKT7 plasmid served as a negative control. The constructs were transformed into bakers’ yeast (*Saccharomyces cerevisiae*) strain Y2HGold (Clontech) following the “Yeast Transformation System 2” protocol provided by the manufacturer. The pCL1 transformants were incubated on SD/leucine dropout media, while the pGBKT7 and pGBKT7-CmMYB2 transformants were incubated on SD/tryptophan dropout media. After culturing at 30 °C for three days, the transformed cell lines were transferred to SD histidine and adenine dropout media.

Luminescence assays were used to detect the transactivation activity of *CmMYB2* in *A*. *thaliana* mesophyll protoplasts. The pENTR1A-*CmMYB2* construct was previously transformed into p*35S::GAL4DB* vectors using a reaction based on LR Clonase II. Transient expression of the transgene was achieved as described previously^36^. A 7.5 μg aliquot of either p*35S::GAL4DB-AtARF5* (positive control), p*35S::GAL4DB* (negative control) or p*35S::GAL4DB-CmMYB2* (test construct) was introduced into the protoplasts. A 7.5 μg aliquot of the luciferase (LUC) reporter construct *GAL4-LUC* was then cotransformed into *A*. *thaliana* mesophyll protoplasts. LUC assays were performed as described previously^37^, except that D-luciferin was replaced by ViviRen Live Cell substrate (www.goldbio.com/). *A*. *thaliana* mesophyll protoplasts were cultivated in plates for 16 h in the light at 23 °C. A low-light cooled CCD imaging apparatus (DU934P, Andor, UK) was used to capture LUC images from a 96-well plate. The LUC activity was measured in 10 s intervals, and the luminescence counts were quantified using a 20/20n luminometer (Turner Biosystems Inc., Sunnyvale, CA, USA). Three independent experiments were performed for each assay.

### Generation and characterization of chrysanthemum transgenics

The *CmMYB2* open reading frame sequence was amplified using primer pair CmMYB2-pBIG-F/R (details given in Table S1), which has a *BamHI* recognition site at its 5′ end and a *SacI* recognition site at its 3′ end, and the resulting amplicon was inserted into a pBIG vector (in which the restriction enzyme sites were reconstructed)^38^. The primer sequences required for the *CmMYB2* RNAi fragment were retrieved from wmd3.weigelworld.org. The fragments required to knock down *CmMYB2* by RNAi were obtained by following an artificial microRNA cloning protocol (wmd3.weigelworld.org/cgi-bin/webapp^39^) and were inserted adjacent to the plasmid’s *Bam*HI and *Sma*I recognition sites (Table S1); the plasmid was subsequently transferred into the pBIG vector. The overexpression and RNAi transgenes were inserted into *Agrobacterium tumefaciens* EHA105 using the freezing transformation method, after which the transformed bacteria were inserted into Jinba plants via *Agrobacterium*-mediated transformation^40^. The growth and development of the two most effective overexpressing (OX-1 and -2) and knockdown (RNAi-1 and -2) plants were assessed at various time points. Flower bud differentiation was divided into five stages: the FBD stage, visible color (VC) stage, EO stage, open-flower (OF) stage and senescent-flower (SF) stage.

### RNA-seq analysis

OX-1, RNAi-2, and WT plants were grown under controlled conditions (23°C, 60% relative humidity, and a 16 h photoperiod provided by 150 μmol m^−2^ s^−1^ illumination) for four weeks. Their third fully expanded leaf and stem apical meristem were harvested at 8 a.m. (after three hours of light exposure), flash frozen in liquid nitrogen and then stored at −80 °C until needed. RNA was extracted from these explants using RNAiso reagent (TaKaRa). RNA-seq was performed on pooled RNA formed by combining equal quantities; the sequencing was performed by a HiSeq 2000 device (Illumina, San Diego, CA, USA) housed at the Beijing Genomics Institute (Shenzhen, China). Adapter contamination sequences (match length ≥ 10 bp), low-quality reads and noncalled bases were discarded to obtain a set of clean reads for use in subsequent bioinformatic analyses. The transcriptomes were assembled into contigs using the Trinity program^41^, and the resulting unigenes were annotated using the Kyoto Encyclopedia of Genes and Genomes (KEGG) (www.genome.jp/kegg/kegg1.html), NCBI nonredundant protein (NR) (www.ncbi.nlm.nih.gov/refseq), NCBI nonredundant nucleotide (NT) (www.ncbi.nlm.nih.gov/nuccore), Swiss-Prot (www.uniprot.org), Cluster of Orthologous Groups (COG) (www.ncbi.nlm.nih.gov/COG/) and Gene Ontology (GO) (geneontology.org/page/go-database) databases. Differentially transcribed genes were subjected to both GO functional and KEGG pathway analyses.

### Quantitative real-time PCR (qRT-PCR) analysis

Total RNA was extracted using RNAiso reagent (TaKaRa), treated with DNase to remove contaminating genomic DNA and reverse-transcribed using M-MLV reverse transcriptase. The resulting ss cDNA served as a template for a series of qRT-PCRs in which each reaction mixture consisted of 10 μL of SYBR Premix Ex Taq™ II (TaKaRa), 0.4 μL of each primer (10 μM) and 5 μL of 1 ng/μL template. The chrysanthemum *EF1α* gene (KF305681) served as a reference. The 2^−ΔΔCt^ method was used to calculate the relative abundance of the various transcripts, and each qRT-PCR was carried out in triplicate.

### Quantifying the GA content in chrysanthemum leaves

WT, OX. and RNAi plants were grown under an 8-h photoperiod for 30 days. Three of the youngest, fully opened leaves (0.5 g of fresh tissue) were harvested after they had been exposed to 3 h of light, with each line sampled in triplicate. The method used to determine the leaf GA content has been described previously^23^.

### Yeast two-hybrid assays

Each of the sequences of *CmBBX*24, *CmBBX24boxs*, *CmBBX24box1*, and *CmBBX*24*box*2 were inserted into pGBKT7 vectors (Clontech), while *CmMYB2* was inserted into a pGADT7 vector (Clontech). The primers used are listed in Table S1. The resulting pGBKT7*-CmBBX*24*boxs*, pGBKT7*-CmBBX*24*box*1, and pGBKT7*-CmBBX*24*box*2 constructs were cotransformed together with pGADT7-*CmMYB2* into Y2HGold cells (Clontech) according to the “Yeast Transformation System 2” protocol. The transformed cells were subsequently incubated on tryptophan and leucine SD media and on tryptophan, leucine, histidine and adenine SD media in the presence or absence of X-*α*-gal. pGBKT7-53 and pGADT7-T served as positive controls, and pGBKT7-Lam pGADT7-T served as negative controls.

### BiFC assays

The *CmBBX24* and *CmMYB2* open reading frames were inserted into the pSAT4A-nYFP and pSAT4A-*cYFP* vectors, respectively^42^. The primers used are listed in Table S1. The resulting constructs and the p*35S::D53-RFP* nuclear marker^43^ were transformed into onion epidermal cells using a PDS-1000He-driven particle bombardment device (Bio-Rad), after which the cells were cultivated on MS media in the dark for 16 h at 23 °C. YFP and RFP activity was observed using confocal laser scanning microscopy (LSM780, Zeiss, Oberkochen, Germany).

## Supplementary information

Primer sequences used in this study

Part candidate of proteins interacting with CmMYB2 and CmBBX24
