# Supplementary material for: Regulation of flowering time in chrysanthemum by the R2R3 MYB transcription factor CmMYB2 is associated with changes in gibberellin metabolism
Source: Hortic Res. 2020 Jul 1;7:96. doi: 10.1038/s41438-020-0317-1 (PMC7326907; doi:10.1038/s41438-020-0317-1)
Supplement: Supplementary file 1 — Primer sequences used in this study [file 41438_2020_317_MOESM1_ESM.doc]

**Supplementary Information**

Supplementary Table S1.Primer sequences used in this study

| Primer | Sequences (5'-3') |
| --- | --- |
| CmMYB2-F | ATGGCTACTGTTTCGAAAA |
| CmMYB2-R | GTCTATTTTACTAATCCCAGTC |
| CmMYB2-pBIG-F | GGATCCATGGCTACTGTTTCGAAAA |
| CmMYB2-pBIG-R | GAGCTCGTCTATTTTACTAATCCCAGTC |
| I miR-s | gaTATTTTACTAATCCCAGTCCTtctctcttttgtattcc |
| II miR-a | gaAGGACTGGGATTAGTAAAATAtcaaagagaatcaatga |
| III miR*s | gaAGAACTGGGATTACTAAAATTtcacaggtcgtgatatg |
| IV miR*a | gaAATTTTAGTAATCCCAGTTCTtctacatatatattcct |
| A | CTGCAAGGCGATTAAGTTGGGTAAC |
| B | GCGGATAACAATTTCACACAGGAAACAG |
| CmMYB2-RNAi-F | CGGGATCCGAATTCCTGCAGCCCCAAAC |
| CmMYB2-RNAi-R | CCCCCGGGCCCCATGGCGATGCCTTA |
| CmMYB2-pENTR1A-F | GCGTCGACATGGCTACTGTTTCGAAAAAA |
| CmMYB2-pENTR1A-R | TTGCGGCCGCGAGTCTATTTTACTAATCCCAGTC |
| CmMYB2-AD-F | CCCATATGATGGCTACTGTTTCGAAAA |
| CmMYB2-AD-R | CGGGATCCGTCTATTTTACTAATCCCAGTC |
| CmBBX24box1-BD-F | CGGAATTCATGTTGAAACAAGAGA |
| CmBBX24 box1-BD-R | CGGGATCCAGCTGTGATGCTCAAGTT |
| CmBBX24box2-BD-F | CGGAATTCCACTCTGCCAATCGCGT |
| CmBBX24box2-BD-R | CGGGATCCTGGGTTTGCAGAATGA |
| CmMYB2-pSAT4A-F | TCGAGCTCAATGGCTACTGTTTCGAAAAAAG |
| CmMYB2-pSAT4A-R | CAGGATCCCGTCTATTTTACTAATCCCAGTC |
