# Supplementary material for: Regulation of flowering time in chrysanthemum by the R2R3 MYB transcription factor CmMYB2 is associated with changes in gibberellin metabolism
Source: Hortic Res. 2020 Jul 1;7:96. doi: 10.1038/s41438-020-0317-1 (PMC7326907; doi:10.1038/s41438-020-0317-1)
Supplement: Supplementary file 2 — Part candidate of proteins interacting with CmMYB2 and CmBBX24 [file 41438_2020_317_MOESM2_ESM.doc]

**Supplementary Information**

**Supplementary Table S2a. Part candidate of proteins interacting with CmMYB2.**

| Encoding protein | Function prediction | No. of Cloning |
| --- | --- | --- |
| CONSTANS interacting protein 3 | Interact with CONSTANS; flowering | 18 |
| 14-3-3 GF14 | Flowering; abiotic stress | 6 |
| CmMYB9A protein | Abiotic stress; flavonoid biosynthesis | 5 |
| COP9 signalosome complex subunit 5b-like | Photomorphogenesis | 4 |
| Wound-responsive family protein (WIN) | Responding to coercion | 1 |
| E3 ubiquitin-protein | Ubiquitin degradation | 2 |

**Supplementary Table S2b. Part candidate of proteins interacting with** CmBBX24.

| Encoding protein | Function prediction | No. of Cloning |
| --- | --- | --- |
| ASPARTIC PROTEASE IN GUARD CELL 1-like | ABA signaling; drought stress | 3 |
| ERD15 | Negative regulator of abscisic acid responses in Arabidopsis | 3 |
| CmMYB9A protein | Abiotic stress; flavonoid biosynthesis | 8 |
| Jasmonate ZIM domain protein | Biotic stress; stamen development, trichome initiation, flower abscission | 2 |
| BBX5/zinc finger protein CONSTANS-LIKE 4-like | Flowering | 1 |
| BBX22/ probable salt tolerance-like protein | Suppress seedling; photomorphogenesis | 3 |
| Nuclear transcription factor Y subunit C-9-like | Interact with CO/CO utilizes NF-Y transcription factor complexes for the activation of FT | 1 |
| BEH4 BES1/BZR1 homolog protein 4-like | BR signaling; flowering | 2 |
| CONSTANS-like protein 1 | Flowering | 3 |
